# Molecular Epidemiology of *Streptococcus pneumoniae* Detected in Hospitalized Pediatric Acute Respiratory Infection Cases in Central Vietnam

**DOI:** 10.3390/pathogens12070943

**Published:** 2023-07-17

**Authors:** Peris Wambugu, Mohammad-Monir Shah, Hien-Anh Nguyen, Kim-Anh Le, Huy-Hoang Le, Hien-Minh Vo, Michiko Toizumi, Minh-Xuan Bui, Duc-Anh Dang, Lay-Myint Yoshida

**Affiliations:** 1 Department of Pediatric Infectious Diseases, Institute of Tropical Medicine, Nagasaki University, Nagasaki 852-8523, Japan; perhizz@gmail.com (P.W.); shah@nagasaki-u.ac.jp (M.-M.S.); toizumi@nagasaki-u.ac.jp (M.T.); 2 Graduate School of Biomedical Sciences, Nagasaki University, Nagasaki 852-8523, Japan; 3 Center for Microbiology Research, Kenya Medical Research Institute, Nairobi 54840-00200, Kenya; 4 Department of Bacteriology, National Institute of Hygiene and Epidemiology, Hanoi 100000, Vietnam; hienanh75@yahoo.com (H.-A.N.); kimanhlt88@gmail.com (K.-A.L.); lehuyhoang2010@gmail.com (H.-H.L.); dangducanh.nihe@gmail.com (D.-A.D.); 5 Department of Pediatrics, Khanh Hoa General Hospital, Nha Trang 650000, Vietnam; doctorhien80@yahoo.com; 6 Khanh Hoa Health Service Department, Nha Trang 650000, Vietnam

**Keywords:** *Streptococcus pneumoniae*, antibiotic resistance, penicillin-binding proteins, macrolide genes, multi-locus typing

## Abstract

*Streptococcus pneumoniae* is the major bacterial pathogen causing high pneumonia morbidity and mortality in children <5 years of age. This study aimed to determine the molecular epidemiology of *S. pneumoniae* detected among hospitalized pediatric ARI cases at Khanh Hoa General Hospital, Nha Trang, Vietnam, from October 2015 to September 2016 (pre-PCV). We performed semi-quantitative culture to isolate *S. pneumoniae*. Serotyping, antimicrobial susceptibility testing, resistance gene detection and multi-locus sequence typing were also performed. During the study period, 1300 cases were enrolled and 413 (31.8%) *S. pneumoniae* were isolated. School attendance, age <3 years old and prior antibiotic use before admission were positively associated with *S. pneumoniae* isolation. Major serotypes were 6A/B (35.9%), 19F (23.7%) and 23F (12.7%), which accounted for 80.3% of vaccine-type pneumococci. High resistance to Clarithromycin, Erythromycin and Clindamycin (86.7%, 85%, 78.2%) and the mutant drug-resistant genes *pbp1A* (98.1%), *pbp2b* (98.8%), *pbp2x* (99.6%) *ermB* (96.6%) and *mefA* (30.3%) were detected. MLST data showed high genetic diversity among the isolates with dominant ST 320 (21.2%) and ST 13223 (19.3%), which were mainly found in Vietnam. Non-typeables accounted for most of the new STs found in the study. Vaccine-type pneumococcus and macrolide resistance were commonly detected among hospitalized pediatric ARI cases.

## 1. Introduction

Acute respiratory infections (ARIs) are the leading causes of childhood morbidity and mortality worldwide. In particular, upper respiratory infections are very common but rarely life-threatening, while lower respiratory infections account for more severe illnesses. *Streptococcus pneumoniae (S. pneumoniae)* is the most common bacterial etiology causing ARIs that resulted in 346,345 deaths in children aged <5 years and 370,288 deaths in children aged 0–14 years old due to lower respiratory infections in 2019 [1,2].

The bacterium usually colonizes the nasopharynx of children at a young age, which is a prerequisite for infection [3,4]. Invasive pneumococcal disease (IPD) occurs when the bacterium enters a sterile site such as blood, cerebrospinal fluid pleural fluid, joint fluid or pericardial fluid. In contrast, non-invasive disease occurs without invasion of the bacteria causing otitis media, sinusitis and bronchitis [5].

IPD is usually caused by about 6–11 serotypes (1, 5, 6A, 6B, 14, 19F, 23F are most common globally) which have guided the design of pneumococcal conjugate vaccines (PCVs) and account for 49–88% of deaths occurring in Africa and Asia [6,7]. *S. pneumoniae* can be classified into >99 serotypes based on type-specific antisera reaction against the capsular polysaccharides, with a 100th serotype 10D from oral *Streptococcus* published so far [8]. PCV protection only occurs against the serotypes in the vaccine. Currently, the World Health Organization (WHO) recommends the use of PCV10 (Synflorix^®^) and PCV13 (Prevnar 13^®^) in children <5 years of age [9].

Vaccination of susceptible populations using the PCVs has played a key role in the reduction in vaccine serotypes, IPD, circulating penicillin non-susceptible and multi-drug-resistant pneumococci shortly after introduction, as seen in studies in the US, England, Wales and Brazil [10,11,12,13]. A reduction in vaccine serotype (VT) pneumococcal carriage was also seen in clinical trials carried out in Vietnam, looking at the most effective and suitable vaccine to implement in the area (either PCV 10 or PCV13) [14]. The widespread use of PCVs, however, alters the serotype distribution, leading to increased non-vaccine serotype (NVT) pneumococci prevalence. This serotype replacement has threatened the effectiveness of the current PCVs in the post-PCV era [8,15].

There is also a worldwide concern about increasing *S. pneumoniae* antimicrobial resistance and the emergence of multi-drug-resistant (MDR) isolates, which have made the treatment of pneumococcal diseases difficult [16]. The intra- and interspecies recombination of *S. pneumoniae* DNA contributes to changes in capsule composition, antibiotic resistance, molecular typing and virulence factors during natural transformation [17,18]. Structural alteration in penicillin-binding proteins (PBP) involved in the synthesis of the cell wall results in penicillin resistance. Of the six *pbp* genes identified in *S. pneumoniae*, three genes, *pbp1a*, *pbp2b* and *pbp2x*, are most often associated with penicillin resistance. Macrolides have also been used for the treatment of pneumococcal infections, and resistance in *S. pneumoniae* usually develops from target modification mediated by the *ermB*-encoded gene that leads to the methylation of 23S ribosomal RNA methylase and the *mefA* gene that encodes efflux pumps [19].

The global spread of antimicrobial resistance shows no sign of slowing down, becoming a significant threat to public health systems not only in developing countries but throughout the world [20]. As of 2022, Vietnam has not introduced PCVs into the routine childhood immunization program. This study, therefore, aimed to determine the pre-PCV pneumococcal carriage rate, serotype distribution, antimicrobial susceptibility and sequence typing of *S. pneumoniae* among hospitalized children having acute respiratory infections in Nha Trang, Vietnam.

## 2. Materials and Methods

### 2.1. Study Design

This was a hospital-based pediatric ARI surveillance from May 2015 to September 2016 in Khanh Hoa General Hospital (KHGH), Nha Trang City, Vietnam. KHGH is the only public provincial hospital that provides inpatient care for sick children in the area, with a bed capacity of 1000 [21].

### 2.2. Patients

The study participants were children more than one month old and under 15 years old who were admitted to the pediatric ward at KHGH during the study period with acute respiratory infections (ARI) defined by cough and/or difficulty in breathing. Written informed consent was received from the parents or guardians of the children. Clinical and demographic data were collected using a structured form that included symptoms and findings at hospitalization and other underlying characteristics. The study hospital setting, target population and patient enrollment at KHGH have been described previously [22].

### 2.3. Specimen Collection

Soon after admission, eligible cases were enrolled in the study and nasopharyngeal swab specimens were collected using a flexible nasopharyngeal flocked swab (Copan, Italy; Cat. No. 503CS01). The swab specimens were then stored in tubes of skim milk–tryptone–glucose–glycerin (STGG) medium and preserved at −80 °C.

### 2.4. Culture, Identification and DNA Extraction of S. pneumoniae

Nasopharyngeal swab samples were cultured and *S. pneumoniae* detection was conducted using a semi-quantitative culture method as previously described [21]. From the *S. pneumoniae* isolates, genomic DNA was extracted manually using the commercial kit (QIAamp DNA Mini kit Qiagen) according to the manufacturer’s instructions and used for the PCR assays.

### 2.5. Serotyping

Isolated *S. pneumoniae* serotyping was performed using a sequential triplex real-time PCR, as described by Pimenta et al. [23], where probes from the CDC *S. pneumoniae* detection protocol for the Asia region were used to target different serotypes. Positive and negative controls were included in all testing. When the serotype of the isolates could not be identified and was *LytA*-positive, the isolate was classified as non-typeable.

### 2.6. Antimicrobial Susceptibility Testing

The antimicrobial agents were selected based on commonly used antibiotics as well as standard treatment guidelines for the treatment of *S. pneumoniae* infection [24]. The agar dilution method was used to determine the minimum inhibitory concentrations (MICs) of the selected antimicrobials, which were Penicillin, Amoxicillin, Amoxicillin/Clavulanate (2:1), Ampicillin, Cefuroxime, Cefotaxime, Cefepime, Imipenem, Azithromycin, Erythromycin, Clarithromycin, Chloramphenicol, Clindamycin, Tetracycline, Ciprofloxacin. The results were interpreted according to the breakpoints of the Clinical and Laboratory Standards Institute (CLSI) criteria (2016) [25] for all antimicrobials except Ciprofloxacin, for which breakpoints from the European Committee on Antimicrobial Susceptibility Testing (EUCAST) 2015 were used [26]. *S. pneumoniae* ATCC 49619 was included as the quality control strain. *S. pneumoniae* isolates were classified as MDR if they were resistant to 3 or more classes of antibiotics [27].

### 2.7. Penicillin and Erythromycin Resistance Genes

PCR was used to detect alterations in the three penicillin-binding protein (PBP) genes that mediate β-lactam resistance (*pbp1a, pbp2b* and *pbp2x*) and genes associated with erythromycin resistance, *ermB* and *mefA*. Details of primers used and PCR conditions are as described in [28,29] and are given in Supplementary Table S1. PBP genotypes are represented as gPRSP (alterations in three *pbp* genes), gPISP (alterations in one or two *pbp*) and gPSSP (absence of alterations in the three *pbp* genes) according to the previously published scheme [30]. For all PCR experiments, positive and negative controls were included.

### 2.8. Multi-Locus Sequence Typing

The internal fragments of seven housekeeping genes, *aroE, gdh, gki, recP, spi, xpt* and *ddl*, were amplified by PCR, sequenced and analyzed using primer pairs described previously [31,32]. Commercial positive control and negative control were included in each experiment. Alleles and sequence types (STs) were determined by the PubMLST database (https://pubmlst.org/spneumoniae/ (accessed on 10 November 2022)). The sequences and STs that were not found in the database were submitted to the curator of the database.

The clustering of related STs was analyzed and visualized by GrapeTree software (stand-alone version), and clonal clusters (CCs) were assigned as groups sharing six identical housekeeping alleles. Profiles of international pneumococcal clones from PMEN (Pneumococcal Molecular Epidemiology Network, http://www.sph.emory.rdu/PMEN (accessed on 20 July 2022) and Global pneumococcal sequencing project, https://www.pneumogen.net/gps/pmen.html (accessed on 20 July 2022) were compared to assess the clones in the study.

### 2.9. Statistical Analysis

Data analysis was carried out using Stata Version 14.0. Demographic, clinical and social characteristics were compared among children colonized with *S. pneumoniae* and those not colonized using logistic regression to calculate odds ratios and 95% confidence intervals (CIs).

## 3. Results

### 3.1. Demographic and Clinical Characteristics of Study Participants

All hospitalized pediatric ARI cases from the target population in Nha Trang City were approached by the study doctors, and over 90% were successfully enrolled. During the study period, 1300 children having acute respiratory infections were enrolled in the study. Overall, more males (780, 60%) than females (520, 40%) were enrolled in the study. The majority of the children came from big families, as 692 (53.2%) had more than four members per household, and 671 (51.6%) attended daycare or school currently. There was no association between smokers in the household and positive pneumococcal detection. The age group with the majority of children was 0–1 years, with 825 (63.5%). Moreover, the number of children found to have pneumonia using the revised WHO Integrated Management of Childhood Illness (IMCI) pneumonia classification [33] was 116 (8.9%). Pneumonia was defined as having tachypnea and/or chest indrawing. Overall, *S. pneumoniae* was detected in 413 (31.8%) children, and in 23.9% of those with clinical pneumonia versus 32.0% of those without it (*p* = 0.551). Moreover, *S. pneumoniae* detection was higher in the age groups of 0–1 and 2–3 years (33.8% and 34.2%, respectively) and lowest among children older than 5 years (11.3%). *S. pneumoniae* carriage was positively associated with children ≤3 years old (OR 2.63, 95% CI 1.69–4.06; for 0–1 years, OR 4.03, 95% CI 1.99–8.16; for 2–3 years, OR 4.10, 95% CI 1.97–8.51) compared to children >5 years, those who attend daycare or school (OR 1.72, 95% CI 1.36–2.18) compared to those who do not, and those who reported antibiotics use prior to admission (OR 1.32, 95% CI 1.01–1.74) compared to those who did not, as shown in Table 1.

### 3.2. Serotyping

**Figure 1:**
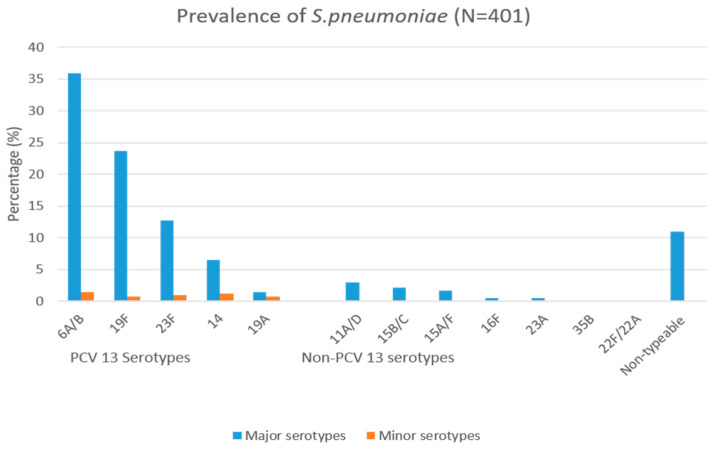
Serotype distribution among 401 *S. pneumoniae* isolates among hospitalized children having acute respiratory infections aged 1 month to <15 years old.

From 413 *S. pneumoniae* isolates, we serotyped 401 isolates, as twelve samples did not grow sufficiently upon revival. Twelve serogroups/serotypes were found. The prevalent VTs were 6A/B (144, 35.9%), 19F (95, 23.7%) and 23F (51, 12.7%). The most prevalent NVTs were 11A/D (12, 3.0%) and 15B/C (9, 2.2%). Serotypes detected together with others, i.e., minor serotypes, amounted to 21 (5.2%). PCV13 serotypes accounted for 80.3% of the serotypes amplified. Those we were unable to serotype were classified as non-typeable (*n* = 44, 11%), as shown in Figure 1.

### 3.3. Antimicrobial Susceptibility Testing

The MICs of 399 *S. pneumoniae* were determined. Fourteen isolates did not grow sufficiently. High antimicrobial resistance was seen for Clarithromycin (346, 86.7%), Erythromycin (339, 85.0%) and Clindamycin (312, 78.2%), while low resistance was observed for Penicillin (1, 0.3%), Ciprofloxacin (2, 0.5%), Cefotaxime (5, 1.3%), Imipenem (41, 10.3%) and Amoxicillin/Clavulanate (43, 10.8%). Resistance to Cefuroxime was the highest among *β*-lactams (71.9%), which is a second-generation cephalosporin, as shown in Table 2. Moreover, 48.6% of the isolates were MDR and the overall resistance to *β*-lactam and Macrolides was 18.4% and 85.9% respectively. The highest seen resistance to Erythromycin and Tetracycline was in serotype 14 (96.2%, 25/26) and 11A/D (91.7%, 11/12), respectively. Comparatively lower resistance rates were seen for Amoxicillin, among which the highest rate was in serotype 19F (34.7%, 33/95) and for Chloramphenicol in 6A/B (66%, 95/144) (Supplementary Table S2).

### 3.4. Penicillin and Erythromycin Resistance Genes

A total of 267 *S. pneumoniae* isolates, isolated between the 2015 October and 2016 May study period, were shipped from Vietnam to Nagasaki University for drug-resistant gene genotyping and MLST analysis. The remaining 132 samples were not able to ship to Nagasaki University in time due to the COVID-19 pandemic situation in Vietnam. A high proportion of PBP gene mutations was present, i.e., *pbp1a* (262, 98.1%), *pbp2b* (264, 98.8%) and *pbp2x* (266, 99.6%). The *pbp* genotype that was the most prevalent was the gPRSP (97%), and no isolate was gPSSP. Erythromycin resistance genes *ermB* were found to amount to 258 (96.6%) and *mefA*, 81 (30.3%), while isolates having both genes amounted to 75 (28.1%). Serotype 19F had the highest distribution of *mefA* and also the highest number of isolates having both *ermB + mefA* genes when compared to other serotypes. However, the *ermB* gene (except in 35B) and *pbp* genes were distributed across all serotypes (Supplementary Table S3).

### 3.5. Multi-Locus Sequence Typing

**Figure 2:**
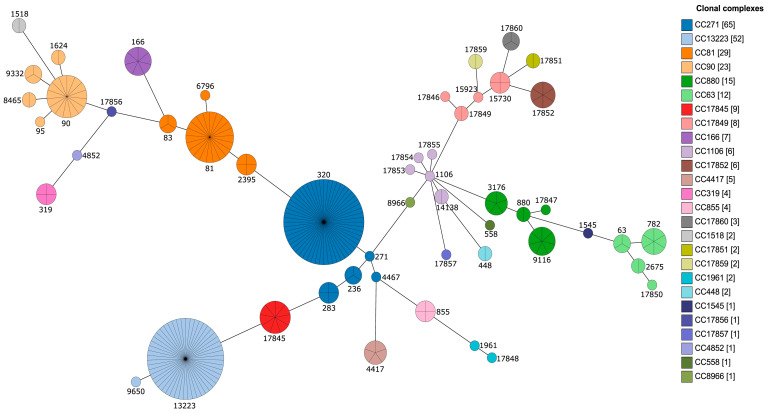
An eBURST diagram showing the pneumococcal sequence (STs) from 264 carriage isolates in Vietnam. CC, clonal complex.

Among 267 *S. pneumoniae* isolates analyzed, three isolates were not successful in the assignment of STs. From 264 isolates, 52 STs were identified by MLST, of which 15 STs (ST 17845, 17846, 17847, 17848, 17849, 17850, 17851, 17852, 17853, 17854, 17855, 17856, 17857, 17859 and 17860) were newly assigned, as shown in Figure 2. The predominant STs were 320 (56, 21.2%), ST13223 (51, 19.3%), ST81 (21, 8%) and ST90 (15, 5.7%).

The genotyped pneumococci clustered into nine CCs and 17 singletons based on the single-locus variant (SLV) in the seven housekeeping genes. The five most prevalent CCs were CC90 (5 STs), CC1106 (5 STs) and CC271 (5 STs). Based on PMEN, four international resistance clones containing 42 isolates were identified. These are Spain^23F^-1 (ST81, 21, 8.3%), Spain^6B^-2 (ST90, 15, 5.9%), Taiwan^19F^-14 (ST236, 3, 1.2%) and Sweden^15A^-25 (ST63, 3, 1.2%), as shown in Table 3. The CCs that showed MDR patterns from the pneumococcal isolates were CC13223, CC90 and the singleton CC17860.

The distribution of STs among different serotypes showed that serotype 6A/B was dominant in ST 13223 and ST 90, with high ST diversity, while serotype 19F was majorly in ST 320. Non-typeable isolates had seven varieties of the most newly assigned ST17851 (*n* = 2), ST17852 (*n* = 4), ST17849 (*n* = 2), ST17853 (*n* = 1), ST17855 (*n* = 1), ST17859 (*n* = 1) and ST17860 (*n* = 2), while serotype 14 had nine isolates of newly assigned ST 17845 (Supplementary Table S4). Among isolates having both *ermB* and *mefA* (74, 29.1%), clone Taiwan^19F^-14 (2, 2.7%) was the only one present. Positive *ermB* and negative *mefA* (174, 68.5%) isolates had four clones distributed among them, i.e., Spain^23F^-1 (21, 12.1%), Spain^6B^-2 (15, 8.6%), Sweden^15A^-25 (3, 1.7%) and Taiwan^19F^-14 (1, 0.57%). Lastly, isolates that did not have the *ermB* gene but had the *mefA* gene (5, 2%) had no international clone present.

## 4. Discussion

The study covered the pre-PCV period (October 2015–September 2016) and focused on children with acute respiratory infections admitted to KHGH. From the study subjects, we identified children ≤3 years, those attending daycare or school and those who had taken antibiotics 14 days prior to admission to be positively associated with the detection of *S. pneumoniae*. The study also found high resistance to macrolides and high diversity of sequence types associated with multi-resistant pneumococcal international clones. This is the first comprehensive report on the population structure of *S. pneumoniae* using MLST in the study area.

In our study, *S. pneumoniae* was detected in 413 (31.8%) pediatric ARI cases, of whom 23.9% had clinical pneumonia and 32.0% did not. However, there was no significant difference between those with and without pneumonia, nor in each respiratory or systemic symptom between those with *S. pneumoniae* and those without. *S. pneumoniae* is a common pathogen causing pneumonia in hospitalized children [5]. Therefore, we could regard the detection of *S. pneumoniae* as colonization in many cases, but some may be the cause of respiratory symptoms in the children. A previous study that detected *S. pneumoniae* from nasopharyngeal samples using real-time PCR in children with radiologically confirmed pneumonia (RCP), other lower respiratory infections (LRTIs) and healthy controls reported that although the detection rates were similar in these groups, the median nasopharyngeal bacterial load of *S. pneumoniae* was substantially higher in children with RCP compared to other LRTIs and healthy controls [34]. We did not conduct the *S. pneumoniae* bacteria load real-time PCR testing in this study, so we were not able to confirm this previous finding. On the other hand, it is important to note that this assumption may not hold true in all cases, as reported in a recent Canadian study [35].

The proportion of serotypes detected were mainly those covered by the PCV 13 vaccine. The dominant VTs found in this study, 6A/B (35.9%), 19F (23.7%) and 23F (12.7%), are similar to a review study carried out on healthy children in the South East Asia region from 2001 to 2019, in which they found an overall pneumococcal prevalence of 36% (4139/11,501) and dominance of serotype 6A/B (12.9%), 23F (9.3%) and 19F (10.1%). Moreover, a study in hospitals in the UK and Ireland on community-acquired respiratory infections pre-PCV7 from 1999 to 2007 found the dominating serotypes in the respiratory tract to be 19F (83, 11.1%), 23F (72, 9.6%), 6B (52, 7.1%) and 3 (51, 6.8%) among children and adults [34]. This changed over time following the introduction of PCV, as reported in a study conducted from 2006 to 2018 that saw an overall decrease in all the dominant vaccine serotypes, with the exception of serotype 3 (13, 1.27%) [35]. In 2001, PCV7 was approved in Europe, and from 2006 to 2008, it was introduced into the national immunization programs of many European countries. In 2009, higher-valent PCVs were adopted, i.e., PCV10 and PCV13, replacing PCV7 from 2009 to 2011. This led to changes in circulating serotypes in the European region to the most frequently isolated 24F, 22F, 8 and 15A in countries that use PCV13, and serotypes 19A and 3 in countries that use PCV10 [36]. This highlights the need to continuously monitor the evolving serotypes and their impact on currently available pneumococcal vaccines.

Our study isolated NVTs 11A/D (3%), 15B/C (2.2%) and 15A/F (1.7%), which have been reported previously both in pre-PCV and post-PCV implementation. A review that focused on serotype distribution causing IPD in the post-PCV era from 2000 to 2015, reported that the predominant non-PCV13 serotypes seen were 22F, 12F, 33F, 24F, 15C, 15B, 23B, 10A and 38, which changed by rank per region of the world [37]. In addition, a study in Japan reported an 8% to 48.1% increase in NVTs from 2010 to 2017 [30], whereas in Denmark in 2014, 71% of IPD was caused by NVTs after the introduction of PCV vaccines. This increase was also reported in the US in 2011 where common NVTs were 33F, 22F, 12, 15B, 15C, 23A and 11 [38]. This trend is worrying, as serotype replacement and switching as a result of vaccine pressure, host dynamics or the environment have the ability to render the current vaccines ineffective. There is an increasing need to develop serotype-independent effective vaccines that can cover vulnerable populations and are capable of targeting all invasive *S. pneumoniae* in the future. Vietnam, however, has yet to start using the currently available PCVs. Based on our study data, further surveillance is necessary to see how much pneumococcal carriage and antibiotic resistance would decrease after PCV introduction in the study area.

In Vietnam, factors that may have led to the high antimicrobial resistance include unrestricted access to antimicrobials through private pharmacies and their irrational use in farming and animal rearing, leading to constant selective pressure and the spread of the global pandemic multi-resistant clones Taiwan^19F^-14 and Spain^23F^-1 from as early as 1993, which when seen are not easily lost [39,40]. A review article that reported on antimicrobial resistance in pediatric pneumococcal isolates globally from 2000 to 2020, before and after the introduction of any PCV, found that there were absolute reductions in resistant isolates over 10 years after any PCV implementation apart from resistance to macrolides and tetracycline [41]. Furthermore, a study conducted from 2016 to 2017 on clinical isolates causing IPD from 13 hospitals in Vietnam reported macrolide resistance of 94% (1234/1317) [42]. This would indicate the importance of following the resistance to macrolides and tetracycline even after PCV implementation.

Our study also found a low rate of β-lactam resistance and a high proportion of PBP gene mutations, signifying that *pbp* mutations did not mainly confer resistance to penicillin in our isolates. Fani et al. reported that other factors could be responsible for β-lactam resistance other than altered *pbps* alone [43]. Furthermore, Erythromycin-resistant *S. pneumoniae* genotypes are usually different over various geographic areas. The most common mechanism of resistance is usually *ermB* encoded in many parts of Asia such as Japan, Sri Lanka, China, Taiwan and South Korea as well as in Belgium (91.5%), France (90%), Spain (88.3%), Serbia (82.4%), Poland (80.8%) and Italy (55.8%). Meanwhile, *mefA*-encoded resistance was most common in Greece (66.2%), the US before PCV7, Finland (55.4%), the UK (70.8%), Australia (59.5%) and Germany (53.2%) [44]. Our study showed *ermB* to be the common mode of resistance for Erythromycin. Serotype 19F was found to have more resistance genes compared to other serotypes. This has been seen in other studies, such as one from Tunisia that reported *ermB + mefA* to be 64% in 19F isolates and one in Korea that reported 57% of both genes in 19F [45,46].

The sequence types predominant in the study were ST320, ST13223, ST81 and ST90. They were mostly found within MDR CCs and within three international clones, Spain^23F^-1, Spain^6B^-2 and Taiwan^19F^-14, responsible for the spread of MDR isolates globally, including in Vietnam [47,48]. Most of these isolates were resistant to Erythromycin, Tetracycline and Cefuroxime. CC271 isolates were associated with serotype 19F, which has been reported in North America and several other Asian countries [49,50,51,52]. However, the clonal spread of CC271 and serotype replacement within the CC has been reported, which is of great concern in the control of VTs [53]. An MDR CC that was uniquely predominant in this study was CC13223. This CC was in serotype 6A/B and resistant to Erythromycin, Cefuroxime, Chloramphenicol and Tetracycline. Non-typeable isolates from this study led to the discovery of more new ST varieties (seven different types) compared to other serotypes. This could highlight the importance of surveillance of non-typeable pneumococci for designing future pneumococcal vaccines as well as understanding the evolution of *S. pneumoniae*. In terms of serotyping, antimicrobial susceptibility testing and multi-locus sequence typing results, there was no major difference in trends of *S. pneumoniae* between the groups with or without pneumonia. The study also shows that apart from vaccine pressure when introduced, other factors such as antibiotic use, mobile genetic elements, capsule switching and age at colonization could play a critical role in the spread of MDR serotypes in PCV-naïve populations.

The study encountered some limitations; the surveillance data only covered one year at one hospital and only hospitalized pediatric ARI cases, making it difficult to generalize the results to the general population. Future studies with the inclusion of community samples will be important. Resistance genes and MLST analysis were carried out on 267 isolates from a total of 399. This, however, was not a shortcoming of the study, as there was little difference in serotype distribution and susceptibility testing results between the isolates that were analyzed and those that were not. We believe that the results from the 267 isolates from the October 2015 to May 2016 period can be representative of the overall isolates collected in this study. We were also unable to distinguish different serotypes within serogroups such as 6A/B, 11A/D and 15A/F and could not confirm non-typeables due to limitations of the serotyping method used. However, the main serotypes that are included in PCVs were identified.

## 5. Conclusions

Children three years old or younger, those attending daycare or school and those who used antibiotics 14 days prior to hospital admission were positively associated with pneumococcal colonization. PCV introduction would be beneficial in this area as high rates of VTs were detected in hospitalized pediatric ARI cases and were associated with antibiotic resistance.

## Figures and Tables

**Table 1:** Clinical and demographic characteristics of children enrolled in hospital-based surveillance from October 2015 to September 2016.

Characteristic	All *n* (%)	Pneumococcal +ve *n* (%)	Pneumococcal −ve *n* (%)	Odds Ratio (95% CI)
Total (*n*)	1300	413 (31.8)	887 (68.2)	
Age				
0–1 yr.	825 (63.5)	279 (33.8)	546 (66.2)	**4.03 (1.99–8.16)**
2–3 yrs.	316 (24.3)	108 (34.2)	208 (65.8)	**4.10 (1.97–8.51)**
4–5 yrs.	79 (6.1)	17 (21.5)	62 (78.5)	2.16 (0.90–5.20)
>5 yrs.	80 (6.2)	9 (11.3)	71 (88.7)	Reference
Sex				
Male	780 (60.0)	250 (32.1)	530 (67.9)	1.03 (0.81–1.31)
Female	520 (40.0)	163 (31.4)	357 (68.6)	Reference
Day-care or school attendance				
Yes	671 (51.6)	251 (37.4)	420 (62.6)	**1.72 (1.36–2.18)**
No	629 (48.4)	162 (25.8)	467 (74.2)	Reference
Prior antibiotic use ^a^				
Yes	950 (73.1)	317 (33.4)	633 (66.6)	**1.32 (1.01–1.74)**
No	350 (26.9)	96 (27.4)	254 (72.6	Reference
From a big family (>4 members)				
Yes	692 (53.2)	211 (30.5)	481 (69.5)	0.88 (0.69–1.11)
No	608 (46.8)	202 (33.2)	406 (66.8)	Reference
Smoker(s) in the household (*n* = 1273)				
Yes	403 (31.7)	275 (31.6)	595 (68.4)	1.03 (0.94–1.12)
No	870 (68.3)	128 (31.8)	275 (68.2)	Reference
Wheeze				
Yes	666 (51.2)	206 (30.9)	460 (69.1)	0.92 (0.73–1.17)
No	634 (48.8)	207 (32.6)	427 (67.4)	Reference
Cough *				
Yes	1298 (99.8)	412 (31.7)	886 (68.2)	0.47 (0.29–7.45)
No	2 (0.2)	1 (50.0)	1 (50.0)	Reference
Crackle upon lung examination				
Yes	284 (21.8)	88 (31.0)	196 (69.0)	0.95 (0.72–1.27)
No	1016 (78.2)	325 (31.9)	691 (68.0)	Reference
Danger signs ^b^				
Yes	27 (2.1)	10 (37.0)	17 (63.0)	1.27 (0.59–2.75)
No	1273 (97.9)	403 (31.7)	870 (68.3)	Reference
Pneumonia				
Yes	116 (8.9)	34 (29.3)	82 (70.7)	0.88 (0.58–1.33)
No	1184 (91.1)	379 (32.0)	805 (68.0)	Reference
Difficulty breathing ^c^				
Yes	129 (9.9)	38 (29.5)	91 (70.5)	0.89 (0.59–1.32)
No	1171 (90.1)	375 (32.0)	796 (68.0)	Reference
Any virus detection ^d^				
Yes	851 (65.5)	276 (32.4)	575 (67.6)	1.09 (0.85–1.39)
No	449 (34.5)	137 (30.5)	312 (69.5)	Reference
Duration of hospitalization				
Range (min–max)	0–22 days			
0–5 days	894 (68.8)	289 (32.3)	605 (67.7)	Reference
6–10 days	374 (28.8)	118 (31.6)	256 (68.4)	0.96 (0.74–1.25)
>10 days	32 (2.4)	6 (18.8)	26 (81.2)	0.48 (0.19–1.19)

^a^ Those who did not know antibiotic history were added to the group of no antibiotic use 14 days prior to admission. * Fisher’s exact test, Chi-square was used on variables without the sign. ^b^ Variables such as unable to drink, altered consciousness, convulsions, lethargy, poor sucking, toxic appearance, irritability, hypothermia and bulging fontanel were all put together to show the danger signs. ^c^ Included those observed by doctor as having difficulty breathing, those having tachypnea based on respiratory rate and those having chest indrawing. ^d^ Viruses detected from these participants were *influenza A* virus, *influenza B* virus, respiratory syncytial virus, rhino virus, adeno virus, boca virus, human metapneumovirus, human parainfluenza virus (type 1, 2, 3 and 4), human coronavirus (229E and OC43).

**Table 2:** Antimicrobial susceptibility of 399 pneumococcal isolates to 13 common antimicrobial agents.

Antimicrobials	Susceptible *n* (%)	Intermediate *n* (%)	Resistance *n* (%)	MIC (µg/mL) Breakpoints S-R
* Penicillin (parenteral non-meningitis)	394 (98.7)	4 (1)	1 (0.3)	≤2–≥8
Amoxicillin (non-meningitis)	271 (67.9)	66 (16.5)	62 (15.5)	≤2–≥8
Ampicillin	42 (10.5)	281 (70.4)	76 (19.0)	≤0.25–≥8
Amoxicillin/Clavulanate	290 (72.7)	66 (16.5)	43 (10.8)	≤2/1–≥8/4
Cefuroxime (parenteral)	74 (18.5)	38 (9.5)	287 (71.9)	≤0.5–≥2
* Cefotaxime (non-meningitis)	368 (92.2)	26 (6.5)	5 (1.3)	≤1–≥4
Imipenem	232 (58.1)	126 (31.6)	41 (10.3)	≤0.12–≥1
Erythromycin	55 (13.8)	5 (1.3)	339 (85.0)	≤0.25–≥1
Clarithromycin	48 (12.0)	5 (1.3)	346 (86.7)	≤0.25–≥1
Chloramphenicol	270 (67.7)	0	129 (32.3)	≤4–≥8
Clindamycin	84 (21.1)	3 (0.8)	312 (78.2)	≤0.25–≥1
Tetracycline	74 (18.5)	21 (5.3)	304 (76.2)	≤1–≥4
Ciprofloxacin	28 (7.0)	369 (92.5)	2 (0.5)	≤0.25–≥4

* Penicillin and Cefotaxime, CLSI 2016 non-meningitis breakpoints were applied. All were interpreted using CLSI 2016, except Ciprofloxacin, for which EUCAST 2015 was used. MIC, minimum inhibitory concentrations.

**Table 3:** Clonal complexes, sequence types, PMEN, resistance patterns and serotype distribution in 254 *S. pneumoniae* isolates.

CC/Singletons	No. of Isolates (%)	No. of STs	Sequence Types (STs)	PMEN	Resistance Patterns *	Serotypes
CC (9)						
90	23 (8.7)	5	8465, 1624, 90, 9332, 95	Spain^6B^-2	ERY + CXM + TE + C	6A/B
1106	6 (2.2)	5	17855, 17854, 1106, 14138, 17853		ERY + CXM + TE	23F(1), 11A/D (1), 16F(1), NT (3)
271	65 (24.6)	5	236, 283, 320, 4467271	Taiwan^19F^-14	ERY + CXM + TE	19F(62), 14(1), 19A(1), 6A/B(1)
81	29 (11)	4	2395, 81, 6796, 83	Spain^23F^-1	ERY + CXM	23F(26), 15B/C (3)
880	15 (5.7)	4	3176, 880, 17847, 9116		ERY + CXM + TE	23F(13), 19F(1) 11A/D(1)
17849	8 (3)	4	17849, 17846, 1593, 15730		ERY + CXM + TE	19F(1), 6A/B(1) NT (6)
63	12 (4.5)	4	17850, 2675, 63, 782	Sweden^15A^-25	ERY + TE	14(6), 15A/F(6)
13223	52 (19.7)	2	13223, 9650		ERY + CXM + TE + C	6A/B
1961	2 (0.8)	2	1961, 17848		TE	15B/C
Singletons (17)						
319	4 (1.5)	1	singleton		ERY	19A
8966	1 (0.4)	1	singleton		ERY + CXM + TE	22F/A
17845	9 (3.4)	1	singleton		ERY + CXM + TE	14
17856	1 (0.4)	1	singleton		ERY + CXM + TE	6A/B
17857	1 (0.4)	1	singleton		0	15B/C
17851	2 (0.8)	1	singleton		CXM + TE	NT
4852	1 (0.4)	1	singleton		ERY + CXM + TE	19A
166	7 (2.7)	1	singleton		ERY + CXM + TE	11A/D
855	4 (1.5)	1	singleton		ERY + CXM	6A/B
448	2 (0.8)	1	singleton		ERY + CXM	14 (1), 16F (1)
4417	5 (1.9)	1	singleton		ERY + CXM + TE	6A/B
17852	6 (2.3)	1	singleton		ERY + CXM + TE	NT(4), 6A/B(2)
1518	2 (0.8)	1	singleton		ERY + CXM	6A/B
1545	1 (0.4)	1	singleton		0	15B/C
17859	2 (0.8)	1	singleton		ERY + CXM + TE	11A/D, NT
558	1 (0.4)	1	singleton		CXM + TE	35B
17860	3 (1.1)	1	singleton		ERY + CXM + TE + AMP	6A/B(1), NT(2)

* Resistance pattern shown to represent different antimicrobial classes of drugs. ERY: Erythromycin, C: Chloramphenicol, TE: Tetracycline, CXM: Cefuroxime, AMP: Ampicillin, PMEN: Pneumococcal Molecular Epidemiology Network.

## Data Availability

Novel sequences from the study were deposited in the PubMLST database (https://pubmlst.org/spneumoniae/ (accessed on 10 November 2022)).

## Supplementary Materials

The following supporting information can be downloaded at: https://www.mdpi.com/article/10.3390/pathogens12070943/s1, Table S1: Primers used for amplification of penicillin-binding protein genes and macrolide resistance genes. Table S2: Antimicrobial resistance of *S. pneumoniae* against antimicrobial agents in different serotypes. Table S3: Resistance genes among different serotypes. Table S4: Sequence type distribution among different serotypes.
