# Supplementary material for: Molecular Epidemiology of Streptococcus pneumoniae Detected in Hospitalized Pediatric Acute Respiratory Infection Cases in Central Vietnam
Source: Pathogens. 2023 Jul 17;12(7):943. doi: 10.3390/pathogens12070943 (PMC10385502; doi:10.3390/pathogens12070943)
Supplement: Supplementary file 1 [file pathogens-12-00943-s001.zip › pathogens-2472640-supplementary.pdf]

Supplementary material

Table S1: Primers used for amplification of pbp and macrolide resistance genes

Primer name	Sequence (5′-3′)	Base pair (bp)	References
LytA	F-$_{681}$ CAACCGTACAGAATGAAGCGG $_{701}$	319	[1]
	R-$_{999}$ TTATTCGTGCAATACTCGTGCG $_{978}$		[1]
Pbp1a	F$_{2037}$ AAACCGCGACTGGGGATCAAC$_{2057}$	239	[2]
	R$_{2275}$ GGTTGAGCTCGACCTTGTTT $_{2256}$		This study
Pbp2x	F-$_{1255}$ CCAGGTTCCACTATGAAAGTG $_{1275}$	197	[1]
	R-$_{1451}$ ATCCCAACGTTACTTGAGTGT$_{1431}$		[1]
Pbp2b	F-$_{1566}$ CCTA TATGGTCCAAACAGCCT $_{1586}$	147	[1]
	R-$_{1693}$ GGTCAATTC CTGTCGCAGTA $_{1712}$		[1]
MefA	F-$_{180}$ CTGTATGGAG CTACCTGTCTGG $_{199}$	296	[1]
	R-$_{581}$ CCCAGCTTAGGTATACGTAC $_{562}$		[1]
ErmB	F-$_{721}$ CGTACCTTGGATATT CACCG $_{740}$	224	[1]
	R-$_{944}$ GTAAACAGTTGACGATATTCT CG $_{922}$		[1]

Table S2: Antimicrobial resistance of S.pneumoniae against antimicrobial agents in different serotypes among 401 isolates

Serotype		AMP[1] n (%)	CXM[1] n (%)	CAM[1] n (%)	ERY[1] n (%)	C[1] n (%)	TE[1] n (%)
6A/B	144	21 (14.6)	119 (82.6)	132 (91.7)	130 (90.3)	95 (66)	125 (86.8)
19F	95	33 (34.7)	74 (77.9)	89 (93.7)	87 (91.6)	4 (4.2)	72 (75.8)
23F	51	4 (7.8)	29 (56.9)	28 (54.9)	31 (60.8)	18 (35.3)	25 (49)
14	26	6 (23.1)	18 (69.2)	26 (100)	25 (96.2)	1 (3.8)	20 (76.9)
19A	6	0	1 (16.7)	4 (66.7)	4 (66.7)	0	2 (33.3)
11A/D	12	4 (33.3)	10 (83.3)	12 (100)	10 (83.3)	0	11 (91.7)
15B/C	9	1 (11.1)	5 (55.6)	6 (66.7)	5 (55.6)	2 (22.2)	6 (66.7)
15A/F	7	0	0	6 (85.7)	5 (71.4)	0	4 (57.1)
16F	3	0	2 (66.7)	2 (66.7)	3 (100)	0	2 (66.7)
23A	2	0	0	1 (50)	1 (50)	0	1 (50)
22F/22A	1	0	1 (100)	1 (100)	1 (100)	0	1 (100)
35B	1	1 (100)	1 (100)	1 (100)	0	0	1 (100)
NT[2]	44	6 (13.6)	27 (61.4)	38 (86.4)	37 (84.1)	8 (18.2)	34 (77.3)

[1]AMP: Amoxicillin, CXM: Cefuroxime, CAM: Clarithromycin, ERY: Erythromycin, C: Chloramphenicol, TE: Tetracycline, [2]NT: Non-typeable. Ampicillin resistance was highest in serotype 19F. While 11A/D and 6A/B showed highest resistance to Tetracycline and Chloramphenicol respectively

Table S3: Resistance genes in different serotypes among 264 isolates

Resistance gene	6A/B n (%)	19F n (%)	23F n (%)	14 n (%)	19A n (%)	11A/D n (%)	15B/C n (%)	15A/F n (%)	22F/A n (%)	35 n (%)	Non-typeable n (%)
N	92	64	40	17	6	10	7	6	1	1	20
ermB	90 (97.8)	64 (100)	40 (100)	16 (94.1)	6 (100)	10 (100)	5 (71.4)	6 (100)	1 (100)	0	18 (90)
mefA	2 (2.2)	61 (95.3)	0	11 (64.7)	1 (16.7)	1 (10)	1 (14.3)	0	0	1 (100)	2 (10)
ermB+ mefA	1 (1.1)	61 (95.3)	0	10 (58.8)	1 (16.7)	1 (10)	0	0	0	0	1 (5)
Pbp1a	92 (100)	63 (98.4)	40 (100)	17 (100)	6 (100)	10 (100)	4 (57.1)	6 (100)	1 (100)	1 (100)	19 (95)
Pbp2b	92 (100)	63 (98.4)	40 (100)	17 (100)	6 (100)	10 (100)	6 (85.7)	5 (83.3)	1 (100)	1 (100)	20 (100)
Pbp2x	92 (100)	64 (100)	40 (100)	17 (100)	6 (100)	10 (100)	6 (85.7)	6 (100)	1 (100)	1 (100)	20 (100)

Distribution of resistance genes across serotypes showed 19F having highest number of isolates having *mefA* and those having both genes *mefA and ermB.*

Table S4: Sequence type distribution among different serotypes in 264 isolates

Serotypes	No. of isolates	CC/Singletons	PMEN	Sequence types (STs),(number)
6A/B	92		Spain^{6B} -2	13223(51),90(15),4417(5),855(4),9332(3),1624(2),8465(2),1518(2),17852(2),95(1),9650(1),17856(1),17860(1),320(1),15730(1)
19F	64		Taiwan^{19F} -14	320(53),236(3),271(1),283(4),4467(1),9116(1),17846(1)
23F	40		Spain^{23F} -1	81(21),880(2),2395(4),3176(5),6796(1),9116(5),17847(1), 17854(1)
14	17			320(1),448(1),782(6),17845 (9)
19A	6			319(4),320(1),4852(1)
11A/D	10			166(7),14138(1),9116(1), 17859(1)
15B/C	7			83(3),1545(1),1961(1),17848(1),17857(1)
15A/F	7		Sweden^{15A}-25	63(3),2675(2),17850(1), 17851(1)
16F	3			1106,448,ND*
22F/A	1			8966
35B	1			558

Non-typeable	20	14138(1),15730(3),15923(1),17849(2), 17851(2),17852(4), 17853(1), 17855(1),17859(1),17860(2),ND*(2)

*ND: Not determined due high nucleotide difference in PUBMLST blast search. The distribution of different STs in serotypes detected. Non-typeables contributed the most (13 isolates) to the new STs found in the study

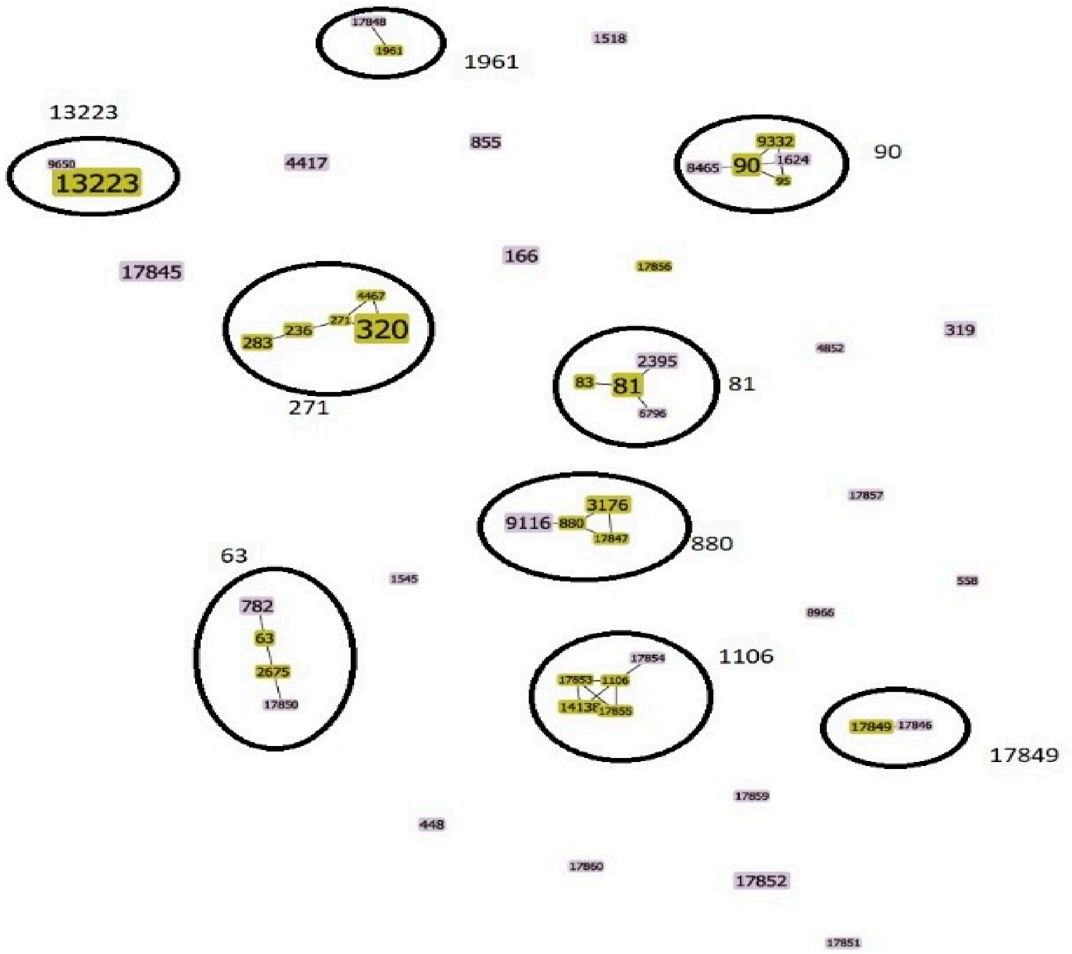

Figure S1: Population snapshot of 264 *S.pneumoniae* isolates

Clusters linked isolates correspond to clonal complexes (CC), CCs are named after predominant ST, and where there is equal distribution of STs in the CC, it is named either isolated ST, STs sharing six (single locus variants- SLVs) were assigned to the same CC, STs not assigned to any CC were designated as singletons

References

1. Nagai, K.; Shibasaki, Y.; Hasegawa, K.; Davies, T.A.; Jacobs, M.R.; Ubukata, K.; Appelbaum, P.C. Evaluation of PCR Primers to Screen for Streptococcus Pneumoniae Isolates and β-Lactam Resistance, and to Detect Common Macrolide Resistance Determinants. *J. Antimicrob. Chemother.* **2001**, *48*, 915–918, doi:10.1093/jac/48.6.915.

2. Ubukata, K.; Chiba, N.; Hasegawa, K.; Kobayashi, R.; Iwata, S.; Sunakawa, K. Antibiotic Susceptibility in Relation to Penicillin-Binding Protein Genes and Serotype Distribution of Streptococcus Pneumoniae Strains Responsible for Meningitis in Japan, 1999 to 2002. *Antimicrob. Agents Chemother.* **2004**, *48*, 1488–1494, doi:10.1128/AAC.48.5.1488-1494.2004.
